# Requirement for endogenous heat shock factor 1 in inducible nitric oxide synthase induction in murine microglia

**DOI:** 10.1186/s12974-015-0406-5

**Published:** 2015-10-14

**Authors:** Chao Huang, Xu Lu, Lijuan Tong, Jili Wang, Wei Zhang, Bo Jiang, Rongrong Yang

**Affiliations:** Department of Pharmacology, School of Pharmacy, Nantong University, #19 Qixiu Road, Nantong, Jiangsu Province 226001 China; Key Laboratory of Inflammation and Molecular Drug Targets of Jiangsu Province, Nantong University, #19 Qixiu Road, Nantong, Jiangsu Province 226001 China; Department of Anesthesiology, Affiliated Hospital of Nantong University, Jiangsu Province, #20Xisi Road, Nantong, Jiangsu Province 226001 China

**Keywords:** Heat shock factor 1, Lipopolysaccharide, Interferon-γ, Inducible nitric oxide synthase, Nuclear factor-κB, Signal transducer and activator of transcription 1

## Abstract

**Background:**

Inducible nitric oxide synthase (iNOS) makes a great contribution to host defense and inflammation. In many settings, lipopolysaccharide (LPS) induces iNOS expression through activation of the inhibitor of κB-α (IκB-α)-nuclear factor-κB (NF-κB) cascade, whereas interferon-γ (IFN-γ) acts through Janus kinase (JAK)-signal transducer and activator of transcription 1 (STAT1) signals. Heat shock factor 1 (HSF1), a major regulator of heat shock protein transcription, has been shown to regulate the production of pro-inflammatory cytokines such as tumor necrosis factor-α (TNF-α) and interleukin-6 (IL-6), but it remains obscure whether and how HSF1 affects iNOS induction.

**Methods:**

Western blot was used to measure the protein expression. The mRNA level was measured by real-time PCR. Silence of HSF1 was achieved by small interfering RNA. Nitric oxide (NO) content and NF-κB binding activity were assayed by commercial kits. Chromatin immunoprecipitation (ChIP) was used to measure the binding activity of NF-κB and STAT1 to iNOS promoters.

**Results:**

HSF1 inhibition or knockdown prevented the LPS- and/or IFN-γ-stimulated iNOS protein expression in cultured microglia. HSF1 inhibition blocked iNOS mRNA transcription. These inhibitory effects of HSF1 inhibition on iNOS expression were confirmed in brain tissues from endotoxemic mice. Further analysis showed that HSF1 inhibition had no effect on IκB-α degradation and NF-κB or STAT1 phosphorylation in LPS/IFN-γ-stimulated cells. The nuclear transport of active NF-κB or STAT1 was also not affected by HSF1 inhibition, but HSF1 inhibition reduced the binding of NF-κB and STAT1 to their DNA elements. In addition, HSF1 inhibition reduced NF-κB and STAT1 bindings to iNOS promoter inside the LPS/IFN-γ-stimulated cells.

**Conclusions:**

This preventing effect of HSF1 inhibition on iNOS mRNA transcription presents the necessary role of HSF1 in iNOS induction.

## Background

Inducible nitric oxide synthase (iNOS or NOS2) is one of the members of the family of nitric oxide synthase (NOS) [1]. Through production of nitric oxide (NO), it plays critical roles in a lot of pathophysiological processes [2]. Under physiological concentrations, iNOS contributes to host defense and inflammation resolution through killing bacteria, tumor cells, and viruses [3–5]. On the other hand, excessive iNOS induces self-damage in disorders associated with inflammation. For example, over-accumulated iNOS has been shown to damage mitochondrial functions and induce cellular apoptosis [6, 7]. The constant production of iNOS renders the vasculature refractory to typical therapies for septic shock such as epinephrine treatment and volume supplementation [8]. Therefore, iNOS induction should be tightly controlled in order to balance the role of iNOS in host defense.

Unlike the constitutively isoform endothelial nitric oxide synthase (eNOS) and neuronal nitric oxide synthase (nNOS), little iNOS is detected in quiescent cells. But in activated cells, iNOS can be induced by various stimuli such as lipopolysaccharide (LPS) and interferon-γ (IFN-γ) [9]. In many settings, LPS induces iNOS gene expression through the classical inhibitor of κB kinase (IKK)-inhibitor of κB α (IκB-α)-nuclear factor κB (NF-κB) signals [10]. In this signaling pathway, LPS first binds with Toll-like receptors leading to IκB-α degradation through the ubiquitin-proteasome system [11]. The removal of IκB-α liberates transcriptional factor NF-κB. The active NF-κB is then free for translocation to the nucleus, where it initiates iNOS gene transcription. Distinct from the mechanism of LPS, IFN-γ triggers iNOS induction through the Janus kinase (JAK)-signal transducer and activator of transcription 1 (STAT1) signals [9, 12]. IFN-γ activates JAK first. JAK phosphorylates transcriptional factor STAT1, which then initiates iNOS gene transcription by binding to the iNOS promoter [13–15]. However, the binding of active NF-κB or STAT1 to the iNOS promoter is not enough to fully initiate iNOS gene transcription. Regulation of iNOS induction may be accomplished by control of IκB-α-NF-κB and/or STAT1 signals upstream of iNOS gene transcription.

Heat shock factor 1 (HSF1) is a major transcriptional factor in the cell with numerous pathophysiological functions. It binds heat shock element (HSE) in the promoter of heat shock proteins (HSPs) with a trimerization form and controls rapid induction of HSPs in cells subjected to heat stresses [16, 17]. It also participates in the regulation of heat shock response, un-associated genes, and pathophysiological processes. For example, HSF1 regulates SPI1/PU.1 expression during macrophage differentiation of monocytes [18] and also glutamate transporter 1 (GLT1) expression in astrocytes [19]. Knocking-out of HSF1 impairs neurogenesis in the dentate gyrus of hippocampus and induces aberrant affective behavior such as increased aggression and depression-like behavior [20]. Both cancer cell’s metabolism and tumorigenesis need the existence of active HSF1 [21, 22]. In inflammatory settings, HSF1 is essential for cells or animals in protection against the toxic effects of bacterial endotoxin through transcriptional repression of pro-inflammatory cytokine genes including tumor necrosis factor-α (TNF-α), interleukin-6 (IL-6), and interleukin-1β (IL-1β) [23–25]. In the present study, inhibition of endogenous HSF1 was found to prevent iNOS induction in LPS- and/or IFN-γ-stimulated murine microglia or endotoxemic brain through attenuation of the bindings of active NF-κB and STAT-1 to iNOS promoter. Our findings, for the first time, show an essential role of endogenous HSF1 in iNOS induction in microglia and establish HSF1 as a potential target for regulation of iNOS gene transcription in inflammatory settings.

## Materials and methods

### Chemicals and reagents

KRIBB11 was purchased from Calbiochem (San Diego, CA, USA). LPS was the product of Sigma (Saint Louis, MO, USA). Antibodies against IκB-α, NF-κB p65, p-NF-κB p65 (Ser536), Histone H2A, glyceral-dehyde-3-phosphate dehydrogenase (GAPDH), p-STAT1 (Tyr701), and STAT1 were purchased from Cell Signaling Technology (Beverly, MA, USA). Antibody against iNOS was purchased from Cell Signaling Technology or Abcam (Cambridge, MA, USA). Protein A/G PLUS-Agarose was the product of Santa Cruz Biotechnology (Santa Cruz, CA, USA). Other related agents were purchased from commercial suppliers. KRIBB11 was dissolved in dimethylsulfoxide (DMSO). The final concentration of DMSO was <0.05 %. All drugs were prepared as stock solutions, and stock solutions were stored at −20 °C.

### Cell preparation

BV-2 cells, kindly provided by Dr. Feng Wu at Nantong University, were grown in DMEM/F12 with 10 % fetal bovine serum (FBS) (Gibco). Mouse primary cultured brain cells were prepared as described previously with some modifications [26]. Briefly, newborn (day 0–1) C57/BL6 mice were decapitated, cortex was then removed and digested with 0.125 % trypsin for 15 min at 37 °C. Followed by trituration and centrifugation at 118 g for 5 min, cells were re-suspended and plated on poly-L-lysine (0.1 mg/mL)-coated culture flasks. The single cell suspension was cultured in DMEM/F12 supplement with 10 % heat-inactivated FBS and 1 % penicillin-streptomycin (100 U/mL). For isolation of primary microglia, the medium was changed to fresh medium after 24 h and replaced every 3 days. After 12 days, mixed cells were shaken gently 2 h at 37 °C, and then the supernatants were collected and plated on the new poly-L-lysine-coated culture flasks. All cells were maintained in a 37 °C incubator containing 95 % air and 5 % CO_2_. After treatment, cell supernatants were collected and frozen at −80 °C for NO detection.

### Cell viability assay

Cell viability was measured using MTT Cell Proliferation and Cytotoxicity Assay Kit (Bi Yuntian Biological Technology Institution, Shanghai, China). Briefly, 5 mg/mL of methylthiazolyldiphenyl tetrazolium bromide was dissolved in prepared MTT-dissolved solutions and kept at −20 °C. After washing with PBS, the cells in plates were added 20 μL of MTT solutions and kept at 37 °C for 4 h. The blue crystals were dissolved in formazan-dissolved solutions, and the absorbance was read at 570 nm.

### Animals and experimental protocol

The use of C57/BL6 male mice was approved by the University Animal Ethics Committee of Nantong University (Permit Number: 2110836). Six- to eight-week-old mice were randomly divided into four groups. In sham- and LPS-treated groups, mice were injected intraperitoneally (i.p.) with 100 μL of saline + DMSO or 100 μL of LPS (25 mg/kg) + DMSO, respectively. In the KRIBB11 (vehicle, DMSO) pretreatment group, mice were administered with a single dose of KRIBB11 (100 μL, 5 mg/kg, i.p). In the KRIBB11 + LPS group, mice were pretreated with a single dose of KRIBB11 (100 μL, 5 mg/kg) 1 h before LPS injection. After that, the brain was immediately excised and frozen in liquid nitrogen, and its additional portions were stored in RNA stabilization reagent RNAlater (Qiagen GmbH, Hilden, Germany) for RNA extraction.

### NO detection

Total nitrite levels were measured with a Griess reagent kit (Invitrogen). The reaction consisted of 150 μL of serum or cell supernatants, 20 μL of Griess Reagent, and 130 μL of de-ionized water. After incubation of the mixture for 30 min at room temperature, nitrite levels were measured at 548 nm using an M2 spectrophotometric microplate reader (Molecular Devices).

### Small interfering RNA (siRNA)

The siRNA oligonucleotides targeting HSF1 and control nonspecific siRNA were purchased from Santa Cruz Biotechnology. In 6-well plates, cells were plated the day before transfection and grown to 30–50 % confluence. The siRNA oligonucleotides (100 nM) were transfected into cells by using Lipofectamine 2000 reagents (Invitrogen). After 48 h of transfection, cells were subjected to further treatments and analysis.

### Real-time PCR

At the end of each treatment, total RNA was isolated from cells or brain tissues using the RNeasy mini kit according to the manufacturer’s instructions (Qiagen, GmbH, Hilden, Germany). First-strand cDNA was generated by reverse transcription of total RNA using the RT system (Promega, Madison, WI, USA). Real-time PCR reactions were conducted with Faststart SYBR Green Master Mix (Roche Molecular Biochemicals) as described in our previous studies [26]. Briefly, 2 μL of diluted cDNA, 0.5 μM primers, 2 mM MgCl_2_, and 1 × FastStart SYBR Green Master mix were employed. The primers for iNOS were 5’-CTC ACT GGG ACA GCA CAG AA-3’ (forward), 5’-TGG TCA AAC TCT TGG GGT TC-3’ (reverse); for 18S rRNA, primers were 5’-GTA ACC CGT TGA ACC CCA TT-3’ (forward), 5’-CCA TCC AAT CGG TAG TAG CG-3’ (reverse). PCR products were detected by monitoring the fluorescence increase of double-stranded DNA-binding dye SYBR Green during amplification. The expression levels of target genes were normalized to the house-keeping gene (18S rRNA). The fold-changes in the target gene expression between experimental groups were expressed as a ratio. Relative gene expression was calculated by the comparative cycle threshold (Ct) method. Melt-curve analysis and agarose gel electrophoresis were used to examine the authenticity of the PCR products.

### NF-κB- and STAT1-binding assays

The nuclei were extracted from BV-2 cells by firstly incubating them in hypotonic buffer containing 10 mM Tris–HCl, pH 7.5, 10 mM NaCl, 1.5 mM MgCl_2_•6H_2_O at 4 °C for 20 min. After homogenization, cell homogenates were spun at 3000 g for 5 min. The supernatants were collected for Western blot. The pellets were recovered, extensively washed, and re-suspended in the nuclear extraction buffer containing 50 mM Tris–HCl, pH 7.4, 150 mM NaCl, 1 % NP-40, 0.25 % sodium deoxycholate, 10 % glycerol, 50 mM NaF, 1 mM Na_3_VO_4_, 5 mM sodium pyrophosphate, protease inhibitors. The NF-κB- or STAT1-binding activity of nuclear extracts was measured with the TransFactor NF-κB colorimetric kit (Clontech, Mountain View) or the DuoSet mouse active STAT1 binding kit (RD Systems, Minneapolis), respectively, according to the manufacturer’s instructions.

### Western blot

To extract the total proteins, cells or tissues were lysed on ice for 30 min in lyses buffer containing 50 mM Tris–HCl, pH 7.4, 1 mM EDTA, 100 mM NaCl, 20 mM NaF, 3 mM Na_3_VO_4_, 1 mM PMSF, 1 % (v/v) NP-40, and protease inhibitor cocktail. The lysates were centrifuged at 12,000 g for 15 min, and the supernatants were recovered. After denaturation, 30–50 μg of proteins were separated on 10 % SDS/PAGE gels and then transferred to nitrocellulose membranes by using a transfer cell system (Bio-Rad, California, USA). After being blocked with 5 % nonfat dried milk powder/Tris-buffered saline Tween-20 for 1 h, membranes were probed with 1:500 primary antibodies against iNOS, IκB-α, p-NF-κB (Ser536), NF-κB, p-STAT-1 (Try701), STAT-1, and Histone H2A or 1:10,000 primary antibodies against GAPDH overnight at 4 °C. Primary antibodies were then removed by washing the membranes 3 times in TBST and incubated further 2 h at room temperature with IRDye 680-labeled secondary antibodies (1:3000–1:5000). Immunoblots were visualized by scanning using Odyssey CLx western blot detection system. For isolation of proteins in the cytoplasm and nucleus, Nucleus Protein Extraction kit was used according to the supplier’s instructions (Bi Yuntian Biological Technology Institution, Shanghai, China). Proteins in the cytoplasm and nucleus were checked by Western blot and were normalized to GAPDH and Histone-H2A, respectively. The band density was quantified using Image J software.

### Chromatin immunoprecipitation (ChIP)

The ChIP experiment was performed as described previously [26]. BV-2 cells were treated with LPS (1 μg/mL) or IFN-γ (20 ng/mL) for 1 h in the absence or presence of KRIBB11. After being incubated in 1 % of formaldehyde solutions on the rocker for 10 min at room temperature, cells were rinsed twice with PBS and lysed for 15 min at 4 °C. After sonication, the lysates were used as DNA input control. The remaining lysates were diluted tenfold with ChIP dilution buffer followed by incubation with NF-κB p65 or p-STAT-1 antibody overnight at 4 °C. Immunoprecipitated complexes were collected using protein A/G Plus-agarose beads. The precipitates were extensively washed and then incubated in the elution buffer containing 1 % SDS and 0.1 M NaHCO_3_ at room temperature for 15 min. Cross-linking of protein-DNA complex was reversed at 65 °C for 4 h. DNA was extracted with the Qiagen PCR purification kit. ChIP assays addressing NF-κB used the following primers: 5′-CAAGCCAGGGTATGTGGTTT-3′ (F), 5′-GCAGCAGCCATCAGGTATTT-3′ (R). ChIP assays for p-STAT1 binding to its IFN-γ-regulated transcription factor STAT1 site on the iNOS promoter used primers 5′-ACACGAGGCTGAGCTGACTT-3′ (F) and 5′-CACACATGGCATGGAATTTT-3′ (R). The resulting products were separated by 2 % agarose gel electrophoresis.

### Statistical analysis

Data were expressed as means ± S.E. One-way analysis of variance (ANOVA) followed by subsequent post hoc analysis was used for the statistical analysis by employing SPSS 11.0 software. Differences were considered significant at *P* < 0.05 or *P* < 0.01.

## Results

### HSF1 inhibition attenuates iNOS induction in LPS/IFN-γ-stimulated microglia

**Fig. 1:**
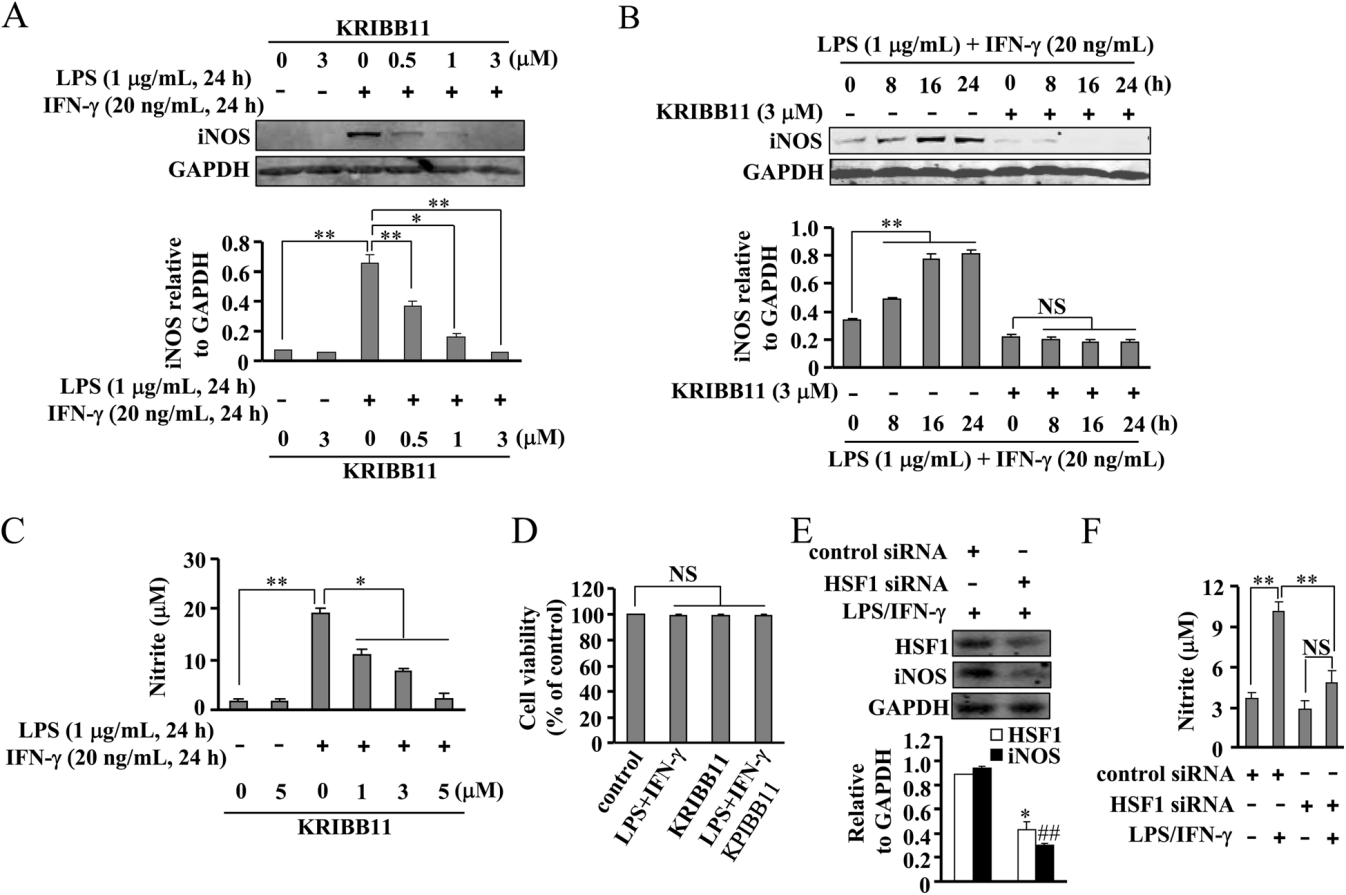
HSF1 inhibition attenuates iNOS induction and NO production in BV-2 cells stimulated by LPS and IFN-γ. **a** *Upper*, representative images showing that KRIBB11 pretreatment (30 min, 3 μM) inhibited iNOS protein expression in cells stimulated by LPS (1 μg/mL) and IFN-γ (20 ng/mL); *lower*, quantitative analysis of iNOS protein expression in cells upon KRIBB11 and/or LPS/IFN-γ treatment (*n* = 3, **P < 0.01 vs. control or LPS/IFN-γ alone-treated group, *P < 0.05 vs. LPS/IFN-γ alone-treated group). **b** *Upper*, representative images showing the time-dependent (8, 16, 24 h) effect of KRIBB11 on LPS/IFN-γ-induced iNOS protein expression; *lower*, a time-course analysis of iNOS protein expression upon KRIBB11 treatment (*n* = 3, **P < 0.01, vs. control group). **c** Quantitative analysis of NO content in KRIBB11- and/or LPS/IFN-γ-treated cells (*n* = 3, *P < 0.05 vs. LPS/IFN-γ alone-treated group, **P < 0.01 vs. control group). **d** Quantitative analysis of the cell viability of cells treated by KRIBB11 and/or LPS/IFN-γ (*n* = 8). **e** *Upper*, effect of HSF1 knockdown on HSF1 and iNOS protein expression in LPS/IFN-γ-stimulated (24 h) cells; *lower*, quantitative analysis of the effect of HSF1 knockdown on HSF1 and iNOS protein expression in LPS/IFN-γ-treated cells (*n* = 5, *P < 0.05, ^##^ *P* < 0.01 vs. control group). **f** Quantitative analysis showing the effect of HSF1 knockdown on NO production after 24 h LPS/IFN-γ treatment (*n* = 5, **P < 0.01, vs. scramble siRNA or scramble siRNA plus LPS/IFN-γ group). All data were shown as mean ± S.E. *NS*, no significance

To determine the role of HSF1 in iNOS induction, BV-2 cells were first treated with LPS and IFN-γ. As anticipated, LPS (1 μg/mL) and IFN-γ (20 ng/mL) treatment induced a significant iNOS protein expression (Fig. 1a, b). Parallel to the increase in iNOS protein levels, increased NO production was measured in cell culture medium from LPS/IFN-γ-stimulated cells (Fig. 1c). Pretreatment of LPS/IFN-γ-stimulated cells with a specific inhibitor of HSF1 KRIBB11 markedly suppressed iNOS expression (Fig. 1a, b). Peak inhibition was observed at a concentration of 3 μM. A time-dependent response curve showed that pretreatment of cells with KRIBB11 (30 min, 3 μM) almost completely blocked the increase in iNOS protein expression at the time points of 8, 16, and 24 h (Fig. 1b). Correspondingly, the level of NO released in the medium also decreased in the KRIBB11-treated cells (Fig. 1c). Cell viability of BV-2 microglia was not affected by KRIBB11 (3 μM) and/or LPS/IFN-γ administration (Fig. 1d). To ensure that the action of HSF1 inhibition on iNOS protein expression was due to HSF1 inhibition rather than any off-target effect, we used siRNA to knockdown endogenous HSF1 content. As shown in Fig. 1e, the HSF1-targeting siRNA, but not the scrambled control siRNA, downregulated endogenous HSF1 content. In line with the results obtained with KRIBB11, HSF1 knockdown significantly attenuated the iNOS protein increase (Fig. 1e) and NO production (Fig. 1f) in the LPS-stimulated cells. These results indicate that HSF1 is essential for iNOS protein expression in the BV-2 cells stimulated by LPS/IFN-γ.

**Fig. 2:**
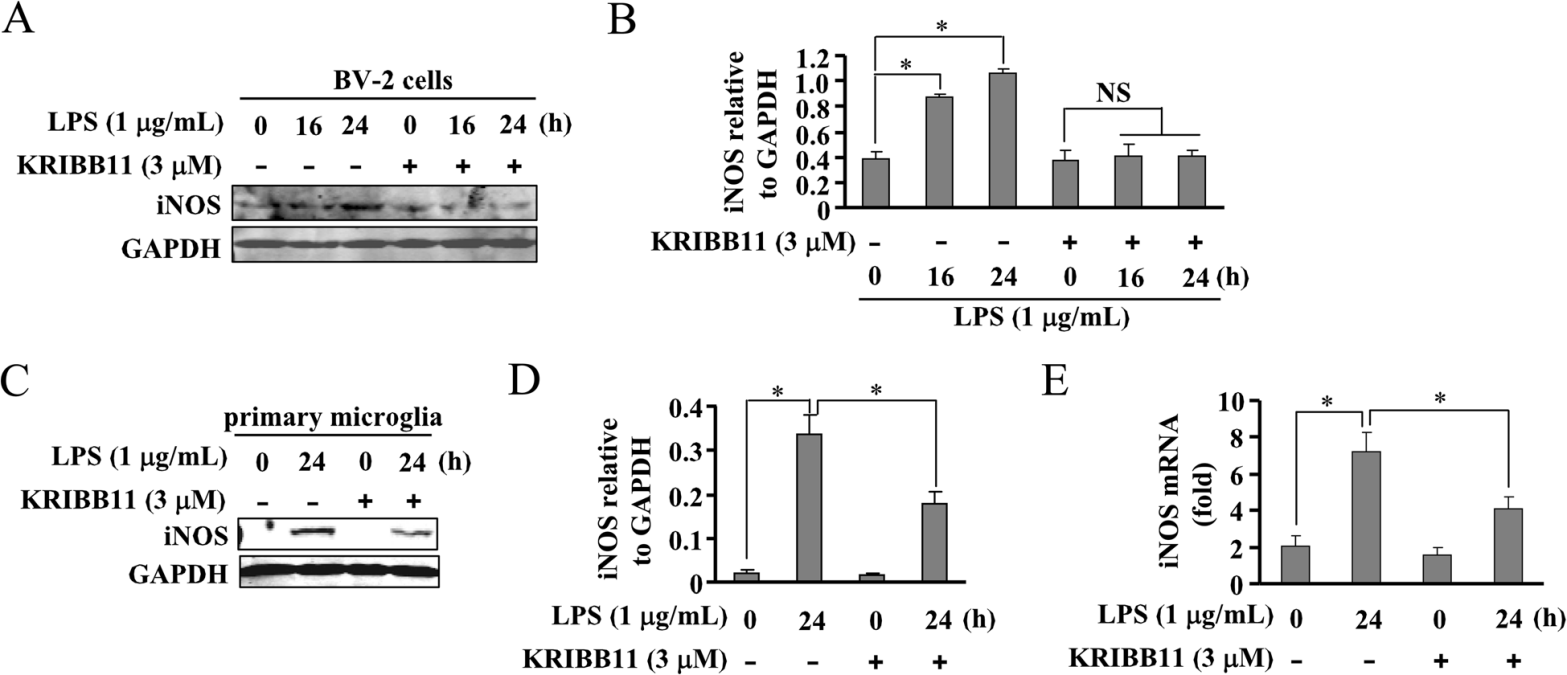
HSF1 inhibition attenuates LPS-induced iNOS expression in BV-2 cells. **a** Effect of KRIBB11 pretreatment (30 min, 3 μM) on iNOS protein expression in LPS-stimulated BV-2 cells. **b** Quantitative analysis of the effect of KRIBB11 on iNOS protein expression in LPS-stimulated cells (*n* = 3, *P < 0.05 vs. control group). **c** Effect of KRIBB11 treatment (3 μM) on iNOS protein expression in LPS-stimulated (24 h) primary microglia. **d** Quantitative analysis of the effect of KRIBB11 on iNOS protein expression in LPS-stimulated primary microglia (*n* = 3, *P < 0.05 vs. control or LPS alone-treated group). **e** Quantitative analysis of the mRNA level of iNOS in primary microglia upon LPS treatment in the absence or presence of KRIBB11 (*n* = 3, *P < 0.05 vs. control or LPS alone-treated group). All data were shown as mean ± S.E. *NS*, no significance

LPS and IFN-γ each can stimulate iNOS gene expression through distinct signaling pathways [9, 10, 12]. Thus, the effect of HSF1 inhibition on iNOS protein expression induced by LPS and IFN-γ were each examined. As shown in Fig. 2, KRIBB11 treatment (3 μM) robustly reduced the LPS-induced increase in iNOS protein expression in both BV-2 cells (Fig. 2a, b) and primary microglia (Fig. 2c, d). The absence of iNOS protein could be due to the deficiency of either gene transcription or protein translation. To distinguish these possibilities, iNOS mRNA formation was measured in the absence or presence of KRIBB11 using real-time PCR. As shown in Fig. 2e, pretreatment of primary microglia with KRIBB11 (3 μM, 30 min) significantly reduced iNOS mRNA formation. These data demonstrate that HSF1 inhibition prevents the LPS-induced iNOS gene transcription in murine microglia.

**Fig. 3:**
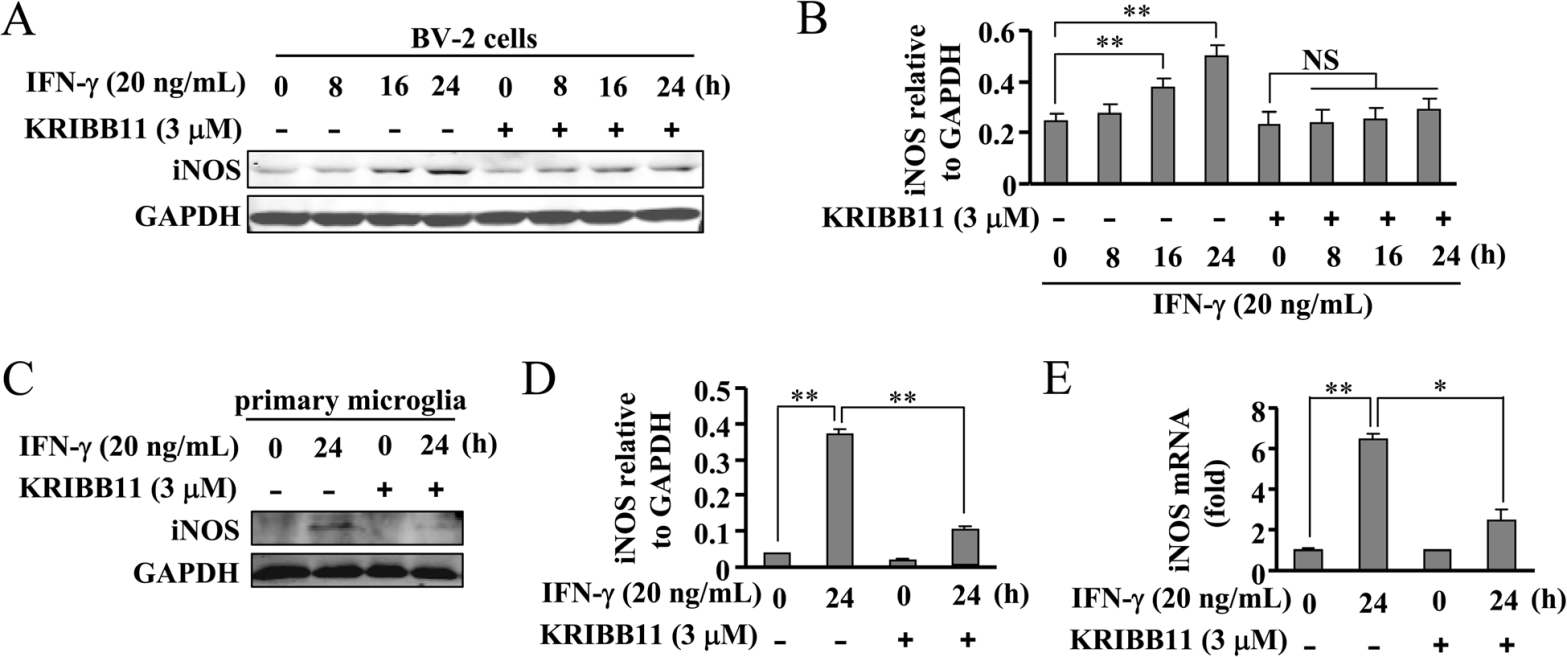
HSF1 inhibition attenuates IFN-γ-induced iNOS expression in BV-2 cells. **a** Effect of KRIBB11 pretreatment (30 min, 3 μM) on iNOS protein expression in IFN-γ-stimulated BV-2 cells. **b** Quantitative analysis of the effect of KRIBB11 on iNOS protein expression in IFN-γ-stimulated cells (*n* = 3, **P < 0.01 vs. control group). **c** Effect of KRIBB11 (30 min, 3 μM) pretreatment on iNOS protein expression in IFN-γ-stimulated (24 h) primary microglia. **d** Quantitative analysis of the effect of KRIBB11 on iNOS protein expression in IFN-γ-stimulated primary microglia (*n* = 3, **P < 0.01 vs. control or IFN-γ alone-treated group). **e** Quantitative analysis of the mRNA level of iNOS in primary microglia upon IFN-γ treatment in the absence or presence of KRIBB11 (*n* = 3, *P < 0.05 vs. IFN-γ alone-treated group, **P < 0.01 vs. control group). All data were shown as mean ± S.E. *NS*, no significance

In BV-2 cells and primary microglia stimulated by IFN-γ, iNOS protein expression was also abolished by KRIBB11 treatment (3 μM) (Fig. 3a–d). Real-time PCR data showed that iNOS mRNA formation was markedly reduced by KRIBB11 in primary microglia (Fig. 3e). Hence, HSF1 inhibition also prevents IFN-γ-stimulated iNOS gene transcription in murine microglia.

### HSF1 inhibition suppresses iNOS expression in brain tissues from endotoxemic mice

**Fig. 4:**
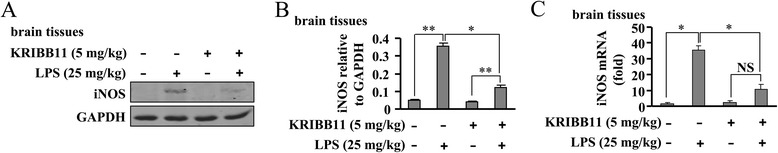
HSF1 inhibition reduces iNOS expression in brains from endotoxemic mice. Mice were divided into sham (saline + DMSO), endotoxemia (LPS 25 mg/kg + DMSO), KRIBB11 alone treatment (5 mg/kg), and LPS + KRIBB11 groups. Mice were sacrificed 20 h after injections and iNOS protein and mRNA levels were measured in brain tissues (**a**–**c**). As shown, KRIBB11 treatment significantly suppressed both iNOS protein (*n* = 3, *P < 0.05 vs. LPS alone-treated group; **P < 0.01 vs. control or KRIBB11 alone-treated group) and mRNA (*n* = 3, *P < 0.05 vs. control or LPS alone-treated group) expressions in brain tissues from endotoxemic mice. All data were shown as mean ± S.E. *NS*, no significance

The above studies revealed the inhibitory role of HSF1 inhibition in iNOS induction in cultured microglia under pro-inflammatory stimuli. To establish the relevance of these biological findings in vivo, the effect of HSF1 inhibition on iNOS expression in brains was investigated using endotoxemic mice. No iNOS was detectable in the brain tissue in the sham group. Injection of LPS into mice induced a marked increase in iNOS protein and mRNA expression in brains (Fig. 4a–c). Consistent with the results observed in cultured microglia, pre-treating mice with KRIBB11 (5 mg/kg, 1 h) markedly prevented the iNOS protein and mRNA expression in brain tissues from endotoxemic mice (Fig. 4a–c).

### HSF1 inhibition does not suppress IκB-α-NF-κB signals in LPS-stimulated BV-2 cells

**Fig. 5:**
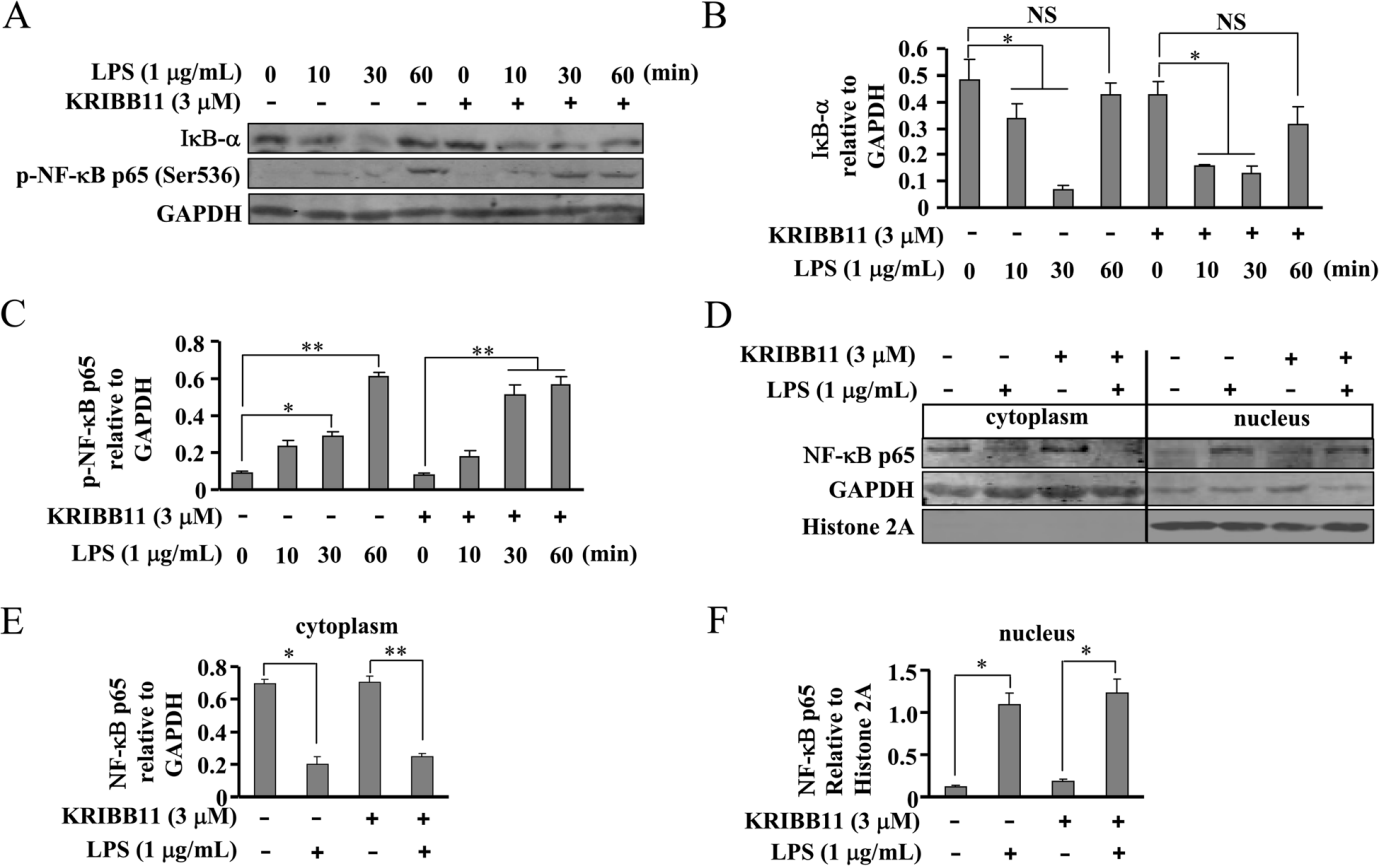
HSF1 inhibition fails to affect LPS-induced IκB-α degradation, NF-κB phosphorylation, and NF-κB nuclear translocation. **a** As shown, LPS-induced increases in IκB-α degradation and NF-κB phosphorylation level were not affected by KRIBB11 treatment (3 μM) in BV-2 cells (*n* = 3). **b**, **c** Quantitative analysis of the effects of KRIBB11 on IκB-α degradation (**b**) and NF-κB phosphorylation (**c**) in LPS-treated cells (*n* = 3, *P < 0.05, **P < 0.01 vs. control or KRIBB11 alone-treated group). **d** Representative images showing the effect of KRIBB11 on NF-κB nuclear translocation. Cells were stimulated with LPS for 30 min, and the levels of NF-κB in the cytoplasm and nucleus were detected. In KRIBB11-treated groups, cells were incubated with KRIBB11 for 30 min prior to LPS stimulation. **e**, **f** Quantitative analysis of NF-κB expression in the cytoplasm (**e**) and nucleus (**f**) in LPS-stimulated cells after KRIBB11 administration (*n* = 3, *P < 0.05 vs. control or KRIBB11 alone-treated group, **P < 0.01 vs. KRIBB11 alone-treated group). All data were shown as mean ± S.E. *NS*, no significance

To explore the mechanism underlying the effect of HSF1 inhibition on iNOS gene transcription, we measured the effect of KRIBB11 on IκB-α–NF-κB signals. As shown in Fig. 5a, b, LPS induced a dramatic increase in IκB-α degradation, and KRIBB11 pretreatment (3 μM, 30 min) did not alter this degradation. It has been reported that the full activation of NF-κB requires its phosphorylation at residue Ser536. Therefore, we determined if HSF1 inhibition affects NF-κB phosphorylation. In accordance with the influence of KRIBB11 on IκB-α degradation, KRIBB11 treatment did not affect NF-κB phosphorylation in LPS-stimulated cells (Fig. 5a, c). Next, we analyzed the change in NF-κB level in the cytoplasm and nucleus of cells stimulated LPS. As shown in Fig. 5d–f, NF-κB was present predominantly in the cytoplasm in un-stimulated cells. LPS treatment resulted in NF-κB nuclear translocation, and this translocation was not counteracted by KRIBB11. The blots were probed with anti-GAPDH (cytoplasmic-specific) and anti-Histone2A (nuclear specific) antibodies to confirm the purity of cytosolic and nuclear fractions (Fig. 5d–f).

### HSF1 inhibition has no significant effect on STAT-1 activation in IFN-γ-stimulated BV-2 cells

**Fig. 6:**
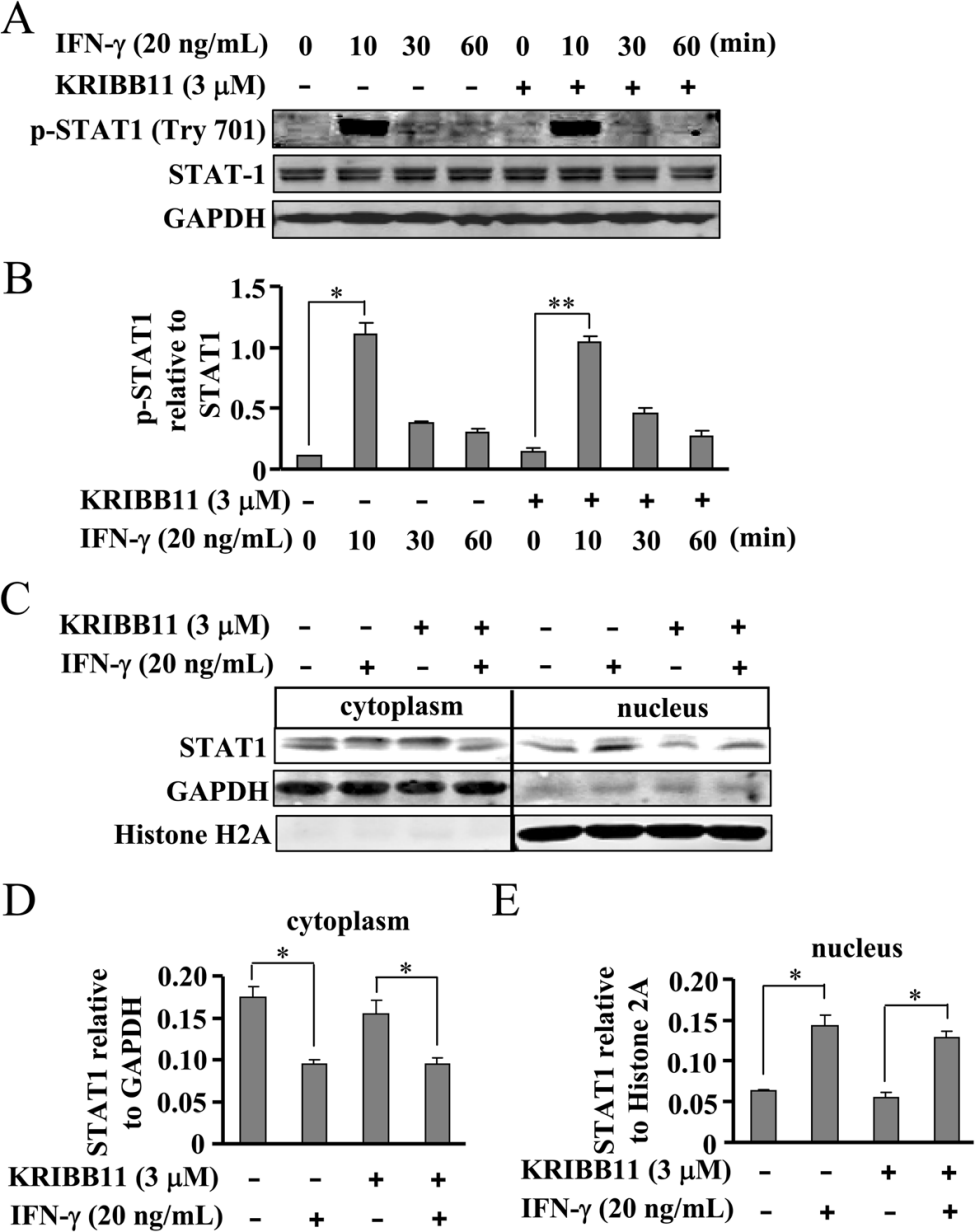
HSF1 inhibition does not inhibit STAT-1 phosphorylation and nuclear translocation in IFN-γ-stimulated cells. **a** As shown, KRIBB11 treatment did not attenuate the increase in STAT1 phosphorylation level in IFN-γ-stimulated BV-2 cells. **b** Quantitative analysis of the effect of KRIBB11 on STAT-1 phosphorylation in IFN-γ-treated cells (*n* = 3, *P < 0.05, **P < 0.01 vs. control group). **c** Representative images showing the effect of KRIBB11 on STAT-1 nuclear translocation. Cells were stimulated with IFN-γ for 30 min and the levels of STAT-1 in the cytoplasm and nucleus were detected. In KRIBB11-treated groups, cells were incubated with KRIBB11 for 30 min prior to IFN-γ stimulation. **d**, **e** Quantitative analysis of STAT-1 expression in the cytoplasm (**d**) and nucleus (**e**) in IFN-γ-stimulated cells after KRIBB11 administration (*n* = 3, *P < 0.05 vs. control or IFN-γ alone-treated group, **P < 0.01 vs. IFN-γ alone-treated group). All data were shown as mean ± S.E

Next, the effect of HSF1 inhibition on STAT1 activation in the process of iNOS expression was studied in IFN-γ-stimulated BV-2 cells. As expected, IFN-γ activated STAT1 by triggering the phosphorylation of STAT1 at Tyr701 residue (Fig. 6a, b). KRIBB11 pretreatment (3 μM, 30 min) affected neither the magnitude nor the time-course of STAT1 activation after IFN-γ stimulation. Short-term exposure to KRIBB11 (3 μM) had no significant effect on cytosolic STAT1 expression (Fig. 6a, b). These data demonstrate that HSF1 inhibition does not block the IFN-γ-stimulated iNOS expression through deactivation of STAT1. To initiate gene transcription, STAT1 also needs to enter into the nucleus. To determine whether HSF1 inhibition affects STAT1 nuclear transport, alterations of the STAT1 level in cytosols and nuclei were measured in cells stimulated in the absence and presence of KRIBB11. As shown in Fig. 6c–e, STAT1 resided in the cytosol of resting cells. IFN-γ stimulation resulted in STAT1 nuclear translocation, and this process was not altered by KRIBB11 treatment (Fig. 6c–e). Thus, HSF1 inhibition had no effect on nuclear transport of STAT1 in IFN-γ-stimulated cells.

### HSF1 inhibition attenuates the DNA-binding activity of NF-κB and STAT1 in LPS- or IFN-γ-stimulated BV-2 cells

**Fig. 7:**
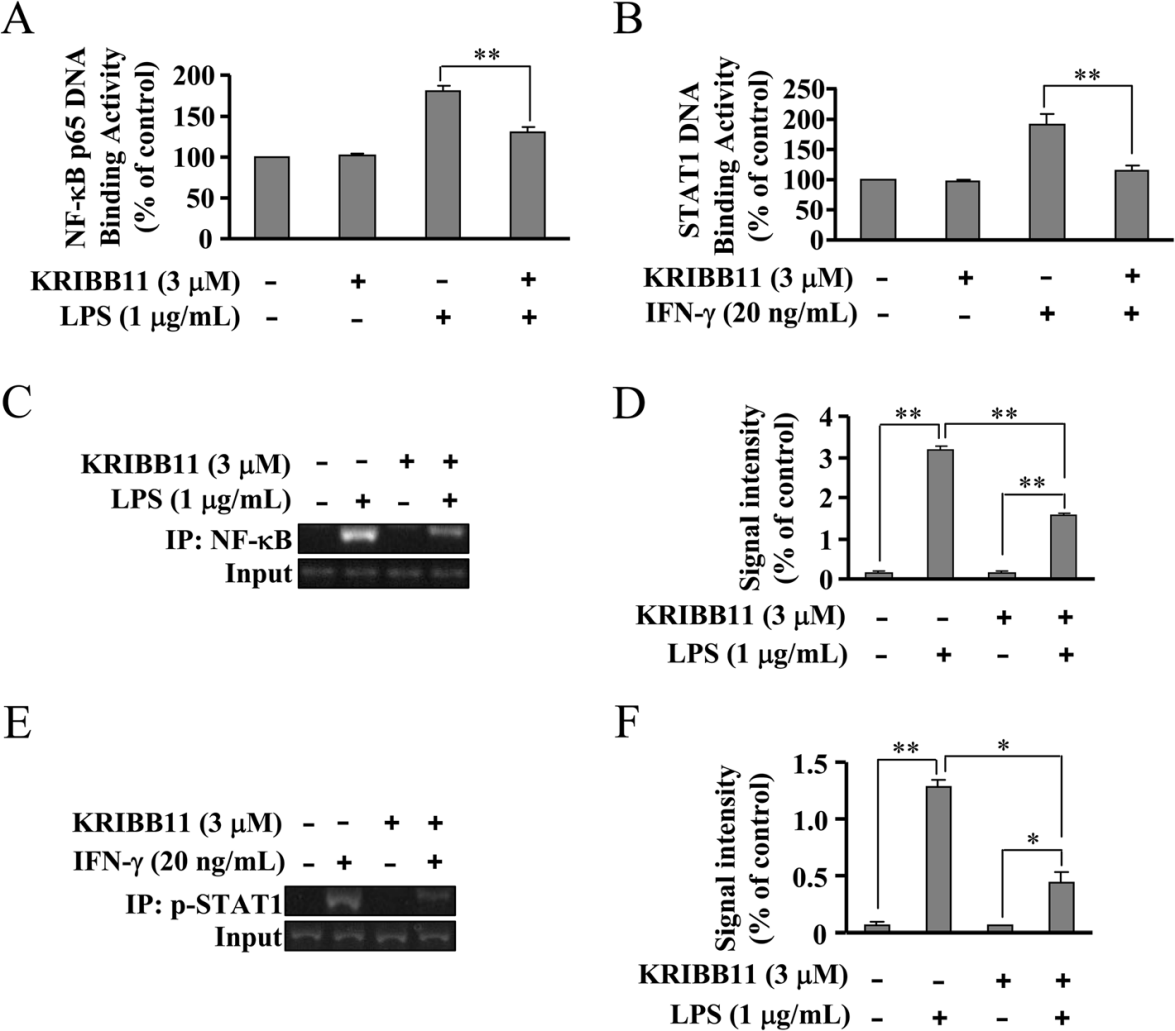
HSF1 inhibition attenuates the binding of active NF-κB and STAT-1 to their DNA elements in iNOS promoters. **a** Quantitative analysis of the effect of KRIBB11 on NF-κB binding to its DNA element. BV-2 cells were stimulated with LPS, and the binding activity of NF-κB p65 was measured in the absence or presence of KRIBB11 (*n* = 5, **P < 0.01 vs. LPS alone-treated group). **b** Quantitative analysis of the effect of KRIBB11 on p-STAT-1 binding to its DNA element in iNOS promoters. Cells were stimulated with IFN-γ, and the binding activity of p-STAT-1 was measured in the absence or presence of KRIBB11 (*n* = 5, **P < 0.01 vs. LPS alone-treated group). **c** Representative ChIP data showing that KRIBB11 markedly reduced NF-κB binding to the iNOS promoter in LPS-stimulated cells. The chromatin was immunoprecipitated using the anti-p65 antibody. **d** Quantitative analysis of the effect of KRIBB11 on the binding of NF-κB to the iNOS promoter in LPS-stimulated cells (*n* = 3, **P < 0.01 vs. control LPS alone-, or KRIBB11 alone-treated group). **e** Representative ChIP data showing that KRIBB11 markedly reduced p-STAT1 binding to the iNOS promoter in IFN-γ-stimulated cells. **f** Quantitative analysis of the effect of KRIBB11 on the binding of p-STAT1 to the iNOS promoter in IFN-γ-stimulated cells (*n* = 3, *P < 0.05 vs. LPS or KRIBB11 alone-treated group, **P < 0.01 vs. control group). All data were shown as mean ± S.E

Since HSF1 inhibition did not appear to affect IκB-α–NF-κB and STAT1 signals, we investigated the possibility that HSF1 inhibition suppresses iNOS transcription by directly interfering with NF-κB and STAT1 binding to their DNA elements in promoters. To explore this possibility, BV-2 cells were stimulated with LPS or IFN-γ to activate NF-κB or STAT1 first. Then, the bindings of active NF-κB and STAT1 with labeled DNA oligos corresponding to their promoters were monitored in the absence and presence of KRIBB11. As shown in Fig. 7a, LPS stimulated a dramatic increase in NF-κB-binding activity in nuclei, and this was blocked by KRIBB11 treatment (3 μM). A similar blocking effect of KRIBB11 was also observed on STAT1-binding activity in the nucleus of IFN-γ-stimulated cells (Fig. 7b). These data suggest that HSF1 inhibition attenuates the binding of active NF-κB and STAT1 with the DNA elements in iNOS promoters. To prove that HSF1 inhibition indeed attenuates the bindings of active NF-κB and STAT1 to the iNOS promoter, we performed ChIP assays on iNOS promoters in stimulated BV-2 cells in the absence or presence of KRIBB11. Results showed that LPS and IFN-γ induced an increased binding of NF-κB or p-STAT1 to the iNOS promoter, and these increases were blocked by KRIBB11 (3 μM) treatment (Fig. 7c–f). Taken together, these results indicated that HSF1 inhibition suppresses the bindings of active NF-κB and STAT1 to iNOS promoters.

## Discussion

The main finding of this study is the identification of the essential role of endogenous HSF1 in iNOS induction in LPS- and/or IFN-γ-stimulated murine microglia. HSF1 belongs to a group of heat shock factor families, which in mammalian cells is composed of three heat shock factors (HSF1, HSF2, and HSF4) [27]. HSF1 has been defined for decades by its ability to coordinate chaperone protein expression and enhance cell survival in the face of heat stress through control of protein transcription in a range of physiological and pathological processes. For example, HSF1 has been shown to mediate the mammalian embryonic development and gametogenesis [28] and cooperates with ErbB2 protein to promote mammary tumorigenesis and metastasis [29]. Genetic studies showed that HSF1 deficiency exacerbates inflammatory responses in cells and animal models [23–25, 28, 30]. Recently, a small-molecule chemical KRIBB11 was found to specifically target HSF1 [31]. The comprehensive analysis indicated that KRIBB11 blocks the induction of HSF1 downstream target proteins such as heat shock protein 27 (Hsp27) and heat shock protein 70 (Hsp70) in cells exposed to heat stress [31]. At the mechanistic level, KRIBB11 inhibited HSF1 function via impairing the recruitment of positive transcription elongation factor b (TEFb) to the Hsp70 promoter [31]. By using KRIBB11, we revealed an essential role of endogenous HSF1 in iNOS induction. HSF1 inhibition with KRIBB11 reduces iNOS induction in murine microglia stimulated with LPS and/or IFN-γ. The action of KRIBB11 was verified by HSF1 knockdown. Further experiments demonstrated that HSF1 inhibition blocks iNOS induction in brains from LPS-stimulated animals. These data strongly indicate that HSF1 is required for iNOS to be maximally induced in cultured cells and endotoxemic mice in response to LPS and/or IFN-γ treatment.

Since excessive iNOS is critical for the development of inflammatory disorders, suppression of iNOS induction by HSF1 inhibition should be beneficial in attenuating the lethal inflammatory responses, but this conclusion appears to be contradicted with the fact that HSF1 deficiency promotes inflammatory responses and disease symptoms [23–25, 28, 30]. One explanation for this contradiction is that HSF1 restricts the overproduction of pro-inflammatory cytokines, such as TNF-α, IL-6, and IL-1β, in macrophages through binding directly with the TNF-α promoter, inducing activating transcription factor 3 (ATF3), or physically interacting with an activator for IL-1β nuclear factor-IL-6 (NF-IL-6), respectively [23–25]. Although the preventing effect of HSF1 on TNF-α, IL-6, and IL-1β production is dependent on HSF1 trimerization [25–27], our recent published data showed that LPS, as a typical inflammatory stimulus, does not induce HSF1 supershift [32] in BV-2 cells, suggesting that the endogenous HSF1 may be involved in iNOS induction in a trimerization-independent and monomer-dependent manner. However, it should be cautious when trying to get such a conclusion because previous studies have shown that the HSF1 monomer cannot bind to the IL-6 promoter in vivo and a little amount of HSF1 trimer in un-stimulated cells helps the IL-6 gene promoter in an open state [24]. Whether HSF1 trimer also helps iNOS gene promoter in an open state remains unclear. More studies should be done to clarify this issue. Taken together, our findings, for the first time, provide novel insight into the pathophysiological role of endogenous HSF1 in iNOS induction.

The mechanistic studies showed that the loss of iNOS protein in HSF1-inhibited cells in due to the lack of iNOS gene transcription. Blockage of gene transcription in inflammatory cells is often due to one or multiple interruptions in the signaling transduction from the stimuli to the corresponding transcriptional cytokines [10]. However, we found no obvious changes in IκB-α–NF-κB signals in LPS-stimulated cells, or STAT1 phosphorylation and nuclear translocation in IFN-γ-stimulated cells. These findings suggest that HSF1 may interfere with signal events downstream of IκB-α degradation and NF-κB or STAT1 nuclear translocation. A significant blockage of the binding of active NF-κB or STAT1 to their DNA elements inside the LPS- or IFN-γ-stimulated cells was observed. Further studies with ChIP assay showed that HSF1 inhibition markedly prevented active NF-κB and STAT1 from bindings to the iNOS promoter. These studies indicate that HSF1 inhibition may directly interrupt the binding of NF-κB and STAT1 to the promoters in the onset of iNOS gene transcription. This may provide a plausible explanation to why iNOS gene transcription was halted, despite that activation of IκB-α–NF-κB and STAT1 signals was not perturbed after HSF1 inhibition, and suggest that the full activation of NF-κB and STAT1 signals triggered by LPS or IFN-γ stimulation in the iNOS promoter needs the presence of endogenous HSF1. In an earlier study, Goldring et al. reported that the rate of iNOS gene transcription was significantly increased in cells exposed to heat shock and LPS, and this increase is associated with HSF1 because the interaction between HSF1 and corresponding iNOS promoter was correspondingly increased under conditions of LPS and LPS plus heat shock treatment [33]. The presence of an anti-HSF-1 antiserum diminished the amount of specific complex formed between HSF1 and iNOS promoter [33]. These evidences strengthen the importance of endogenous HSF1 in iNOS induction. We also noticed that a dominant negative HSF1, which lacks a transactivation domain, competes with NF-κB for binding to this master regulatory control site and so block the NF-κB-mediated induction of MICA by TNF-α [34], suggesting that HSF1 assists the NF-κB to activate gene transcription.

How exactly HSF1 inhibition affects the bindings of NF-κB and STAT1 to their DNA elements in the iNOS promoter remains to be determined. It has been shown that the full initiation of iNOS gene expression not only needs the binding of active NF-κB and/or STAT1, but also needs the involvement of post-translational modifications such as histone acetylation and DNA methylation [35, 36]. The acetylation level is controlled by acetyltransferase (p300) and deacetylase [37, 38], and positively correlated with iNOS gene transcription [35]. DNA methylation, a modification implicated in neurogenesis, synaptic plasticity, learning and memory, aging, and the immune response [39, 40], has been shown to inhibit iNOS gene transcription and negatively controlled by histone acetylation. Given the fact that HSF1 can help the recruitment of p300 acetyltransferase to protein-DNA complex [41], we raise a possibility that HSF1 inhibition prevents iNOS gene transcription likely through increase of DNA methylation. This hypothesis bears further investigations.

## Conclusions

Our present findings for the first time revealed an obligatory role of endogenous HSF1 in iNOS induction in murine microglia. Depending on the inducers, HSF1 helps iNOS gene transcription through promotion of NF-κB and STAT1 bindings to iNOS promoter. Functionally, these findings may establish HSF1 as a potential target for intervening in iNOS induction in the central nervous system (CNS) disorders associated with inflammation.

## Abbreviations

iNOS: inducible NO synthase
LPS: lipopolysaccharide
IκB-α: inhibitor of κB-α
NF-κB: nuclear factor κB
IFN-γ: interferon-γ
STAT1: signal transducers and activators of transcription 1
HSF1: heat shock factor 1
TNF-α: tumor necrosis factor-α
IL-6: interleukin-6
ChIP: chromatin immunoprecipitation
